# Resignations

**Published:** 1863-01

**Authors:** 


### RESIGNATIONS.

PROFESSORS MCQUILLEN and SUESSEROTT, of the Pennsylvania Dental College, have resigned. We do n't know why, but hope it was owing to the "distracted state of the country." Resigning at the beginning of the session was calculated to embarrass the institution; but we are glad to learn, as we have done *by mere accident*, that their places are filled, and that the session is progressing satisfactorily. Dr. GEO. T. BARKER succeeds Prof. S., and a *physician*, whose name is not now remembered, succeeds Prof. Mc Q. W.

